# NMR quality control of fragment libraries for screening

**DOI:** 10.1007/s10858-020-00327-9

**Published:** 2020-06-12

**Authors:** Sridhar Sreeramulu, Christian Richter, Till Kuehn, Kamal Azzaoui, Marcel Jules José Blommers, Rebecca Del Conte, Marco Fragai, Nils Trieloff, Peter Schmieder, Marc Nazaré, Edgar Specker, Vladimir Ivanov, Hartmut Oschkinat, Lucia Banci, Harald Schwalbe

**Affiliations:** 1 Center for Biomolecular Magnetic Resonance (BMRZ), Institute for Organic Chemistry and Chemical Biology, Goethe University, Frankfurt, Germany; 2 Bruker, Fällanden, Switzerland; 3 Saverna Therapeutics, Biel-Benken, Switzerland; 4 Magnetic Resonance Center and Department of Chemistry, University of Florence, Florence, Italy; 5 Department of NMR-Supported Structural Biology, Leibniz-Forschungsinstitut für Molekulare Pharmakologie, Berlin, Germany; 6 Enamine, ENAMINE Ltd., 78 Chervonotkatska Street, Kiev, 02660 Ukraine; 7 German Cancer Consortium (DKTK), Heidelberg, Germany; 8 German Cancer Research Center (DKFZ), Heidelberg, Germany

**Keywords:** Drug discovery, FBDD, Ligands, Fragment, Quality control, Solubility, NMR

## Abstract

Fragment-based screening has evolved as a remarkable approach within the drug discovery process both in the industry and academia. Fragment screening has become a more structure-based approach to inhibitor development, but also towards development of pathway-specific clinical probes. However, it is often witnessed that the availability, immediate and long-term, of a high quality fragment-screening library is still beyond the reach of most academic laboratories. Within iNEXT (Infrastructure for NMR, EM and X-rays for Translational research), a EU-funded Horizon 2020 program, a collection of 782 fragments were assembled utilizing the concept of “poised fragments” with the aim to facilitate downstream synthesis of ligands with high affinity by fragment ligation. Herein, we describe the analytical procedure to assess the quality of this purchased and assembled fragment library by NMR spectroscopy. This quality assessment requires buffer solubility screening, comparison with LC/MS quality control and is supported by state-of-the-art software for high throughput data acquisition and on-the-fly data analysis. Results from the analysis of the library are presented as a prototype of fragment progression through the quality control process.

**Electronic supplementary material:**

The online version of this article (10.1007/s10858-020-00327-9) contains supplementary material, which is available to authorized users.

## Introduction

Fragment-based screening by NMR has evolved as a remarkable approach within the drug discovery process 25 years after the proposal of this approach (Shuker et al. 1996). Since then, fragment-based drug discovery (FBDD) has been an important tool in identifying initial hits against difficult targets and thereby has become one of the foremost and popular methods to be used within the pharmaceutical and biotechnology industry (Baker 2013; Murray and Rees 2009). Vemurafinib from Plexxicon, developed as an anti-melanoma, was the first approved drug using FBDD (Tsai et al. 2008) and followed by several others which are now either approved drugs or in the different phases of clinical trials (Brough et al. 2008; Erlanson et al. 2020; Howard et al. 2009; May et al. 2011; Park et al. 2008; Wang et al. 2010; Woodhead et al. 2010; Wyatt et al. 2008; Zhu et al. 2010).

FBDD has not only become a widely used technology in industry but has been also successfully adopted in academia (Bulfer et al. 2016). Historically, academic institutes have been recognized as screening centers involved in developing tool compounds for genomic studies. In this context large chemical libraries were introduced which further strengthened the academic screening campaigns. From thereon academia has ventured into more challenging translational projects, in particular addressing the “undruggable” target classes and rare diseases. In comparison to high-throughput screening (HTS), the advantage of FBDD has been realized early on. FBDD uses only a few thousand fragments and there by rendering the approach economically affordable. Further, the necessary knowhow and the required infrastructure for performing FBDD (e.g., NMR spectrometers and other instruments) are becoming more and more available at most academic institutes worldwide.

Generally, it has been realized that after a very enthusiastic start of FBDD within academia, soon it becomes an uphill task as these projects enter advanced stages of the drug discovery unlike the industry-based screening campaigns. One of the major struggles within academia-based drug discovery is to develop an initial fragment hit to a lead and drug candidate. In this context, the limited availability of high-quality chemical libraries for academia narrows the chances of discovering specific leads which can be developed into a drug candidate. The former challenge has been overcome by initiating large consortiums involving several academic institutes which work like a “gear-box” and assembles the necessary manpower, materials and instrumentation and strive towards translational research. The latter challenge involving the fragment libraries proves to be one of the major hurdles partly attributed to the fact that the pharmaceutical and biotechnology companies developed and maintained their own specific libraries which were not publicly available. Academic institutes generally resorted to commercial vendors such as Maybridge, Chembridge, Enamine, F2X-universal library, LiverpoolChiroChem, JBS FragXtal screen and the MedChemExpress fragment library and many others (Lepre 2011). Fragment libraries comprive low molecular weight compounds up to a molecular weight of 300 Da. One of their primary requirements is structural diversity to sample a large chemical and structural space. However, most of the commercially available libraries hosts very large sets of fragments with low diversity, issues with solubility or reactivity and therefore may not be suitable for pursuing a screening project within the timeframe of an academic environment. Another challenge which most of these libraries posed is their quality (purity and chemical identity) and also the chemical properties needed for downstream chemistry to pursue either fragment-linking or -growing chemical campaigns with the aim of developing high affinity inhibitors.

We are partners of the iNEXT (Infrastructure for NMR, EM and X-rays for Translational research) consortium, a European facility network to stimulate translational structural biology (iNext Consortium 2018). Within the design of structure-guided drug discovery workflows, iNEXT contributed to FBDD by assembling and validating a fragment library. After careful computational analysis of a large collection of fragments (11,677 in total), a total of 782 fragments were filtered and selected with the aim of “minimum fragments and maximum diversity” to cover a large chemical space and in particular based on the concept of “poised fragments” with the aim to streamline downstream synthesis of more complex and high affinity ligands (Cox et al. 2016). These individual fragments were then purchased from various vendors and assembled.

Quality control of the fragment library is an important and indispensable prior and periodical requirement for pursuing screening measurements (Dalvit et al. 2006). Previously, many researchers have extensively reported several measures to be taken in assessing the quality of a fragment library (Bentley et al. 2018; Dalvit et al. 2006; Gossert and Jahnke 2016; Lepre 2011; Taylor et al. 2018). Importantly, most of these analyses were based on 1D proton NMR spectra. However, there is little information across the literature pertaining to a detailed presentation of the quality control process and were mostly based on a single biophysical technique in determining the quality of the fragments. Further, although considerable research has been devoted to quality assessment of the fragments, rather less attention has been paid to the speed of the assessing protocol. In order to close-in this gap, we present here an integrated approach using commercially available state-of-the-art software Complete Molecular Confidence for quantification (CMC-q) and CMC-assist (CMC-a) developed by the company Bruker, ^1^H-NMR measurements and liquid chromatography-mass spectrometry (LC/MS) for characterization of the integrity and solubility of the fragments. CMC-q and CMC-a efficiently facilitate automated NMR-data acquisition and “on-the-fly” analysis and extract information from complex NMR data, conduct consistency and concentration assessments. Manual cross-validation of the automated NMR software-based quality assurance results together with the LC–MS data was performed for a subset of the fragment library. Approximately 30% of the purchased fragments do not pass the QC and had to be discarded.

## Chemical quality of the library

The design principle of a fragment library holds the key for any successful screening campaign. The iNEXT fragment library was collected using the initial library of “poised fragments” (fragments contain at least one functional group which can be synthesized using a robust, well-characterized reaction). The principle of building and designing such library is described previously (Cox et al. 2016).

**Fig. 1:**
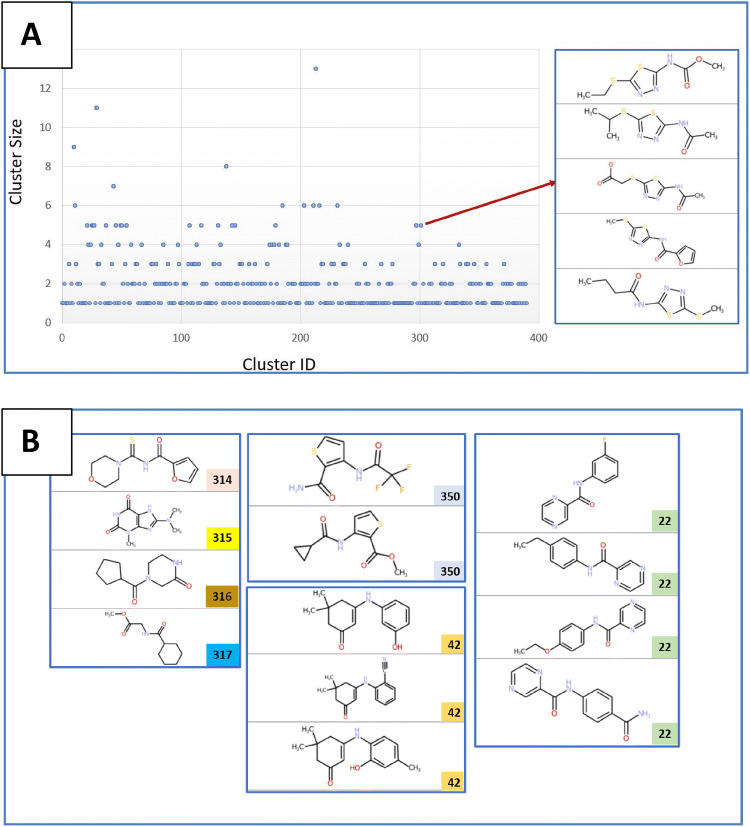
Chemical clustering of the iNEXT-fragment library. **a** Cluster size versus cluster ID (on the top right, an example of compounds belonging to cluster number 301 with five class members). **b** Examples for molecular clusters with 1, 2, 3 and 4 members

In order to estimate the chemical diversity of the library, we performed a molecular clustering analysis of the library using the Knime analytics platform (www.knime.com). The protocol workflow (provided in the Supp. Mat.) of the clustering was performed using the FCFP4 fingerprints (Rogers and Hahn 2010) with a Tanimoto distance matrix calculation followed by a hierarchically clustering of the distance matrix (Threshold of 0.6 to assign a member to a cluster). For the 782 compounds (Supplementary excel sheet), a total of 391 distinguished chemical clusters were found, 198 clusters contain only a single molecule. The clustering analysis suggests a high chemical diversity of the library with a high number of fragments belonging to clusters with 1, 2 or 3 members (Fig. 1a). Examples of molecular clusters are also shown in Fig. 1b and all class IDs and class size are reported in the excel file (Supp. Material).

To assign the drug likeliness of the library, many common molecular descriptors were calculated (see Knime protocol and excel Table in Supp. Mat.). The analysis of molecular weight indicates that around 80% of the fragments are in the range 200–250 Da with hydrogen-bond donor and hydrogen-bond acceptor atoms below three, which clearly satisfies the widely adopted “rule of 3” guide (Congreve et al. 2003; Jhoti et al. 2013).

Since all the fragments comply with the Lipinski rule of 5, we further investigated drug-likeness of the library by calculating the quantitative estimate of drug-likeness (QED) of all the molecules. The concept of QED was introduced by Bickerton et al. (2012) to reflect the underlying distribution of molecular properties and quantify the drug-likeness. The QED with the optimal 1000 weight combinations that give the highest information content (QEDw, mo) was calculated for all the fragments using the equation reported in the above article and the values are listed in Table (Supp. Mat.).

The calculated QED values range from 0.36 to 0.9 with an average value of 0.77. The highest value indicates the most drug-like molecule. 94% of the fragments have a value of QED higher than 0.6 and 44% of the fragments have a value higher than 0.8 indicating a large number of the molecules in the library that can be potentially initiators of a drug candidate (according to QED concept) (Table 1).

**Table 1:** The average values of the molecular descriptors used for the calculation of the QED

Molecular descriptors	Mean
Molecular weight	219.80
SlogP	1.59
Number of hydrogen bonds donors	2.79
Number of hydrogen bonds acceptors	1.27
Topological polar surface area	55.36
Number of rotatable bonds	2.60
Number of aromatic rings	1.43
Number of unwanted substructure alerts	0.17
QED	0.77

The QED equation contains a contribution term used for the number of unwanted substructures that can be related to compound stability, reactivity or toxicity. Using a substructure filter of the unwanted substructures listed in the Bickerton’s article, 111 compounds (14% of the library) were found to have one or more of the unwanted substructures and mostly not very dramatic ones (Table 2). The average QED of this set of compounds is 0.68 showing a low weight given to the unwanted substructure alerts in the calculation.

**Table 2:** Number of compounds with unwanted substructures according to Bickerton et al.

Unwanted substructures (Usub)	Number of compounds with Usub
2-Halo pyridine	2
Acyl hydrazine	7
Aliphatic long chain	1
Aniline	37
Catechol	9
Cumarine	3
Cyanamide	2
Hydantoin	3
Hydrazine	8
Hydroquinone	7
Hydroxamic acid	3
Mercapto-1,3,4- thiadiazole	5
Oxygen–nitrogen single bond	29
Phenol ester	5
Thiocarbonyl group	11
Triple bond	9

This simple analysis of molecular clusters and QED underlines the fact that the iNEXT fragment library is relatively of high quality in its composition and can be used for FBS with a higher chance to identify drug like lead candidate.

## Fragment purchase, stock preparation and storage

A carefully crafted library is a prerequisite for its durability and progression of over several screening campaigns. Typically, there could arise several practical scenarios during the purchase, stock solution preparation and assembly of compounds (Lepre 2011). In general, if the vendor provides a certificate with the exact amount of compound delivered in the vial, one could directly add the exact volume of solvent to attain a desired concentration and avoid the much laborious procedure of weighing and dissolving. DMSO, although being a mild oxidant of some compounds (Prochazkova et al. 2012), is in general the solvent of choice for the preparation of the stock solutions. Typical storage conditions for the fragment libraries is between 4 and − 20 °C in order to avoid any degradation of the compounds over time. However, repeated freeze–thaw cycles can result in degradation of some compounds and also DMSO being hygroscopic can introduce atmospheric water into the stock solutions, thus varying the stock concentration. Addition of 10% water prevents the freezing of the DMSO solution at 4 °C and thus overcomes the freeze–thaw problems (Gossert and Jahnke 2016). Considering all of the above facts, the iNEXT library was assembled by purchasing selected fragments from several vendors, which were dissolved and stored as 50 mM stock solutions in a mixture of 90% *d*_*6*_-DMSO and 10% D_2_O. Freshly prepared stocks were dispensed and stored at 4 °C in V-bottom 0.75 ml 2D-barcoded tubes (Matrix Cat. No 3731) covered with SepraSeal septum caps (Matrix Cat. No 4463).

## Fragment characterization: methods of choice for quality control

A careful determination of ligand integrity and solubility under the given condition is one of the prime aims within the quality control of the fragment library. Some of the measures taken to ensure the quality of the fragments have been elegantly discussed and described previously in the literature (Gossert and Jahnke 2016). We used an integrated approach utilizing the software Complete Molecular Confidence for quantification (CMC-q) and CMC-a, ^1^H-NMR experiments and liquid chromatography-mass spectrometry for characterization of the integrity and solubility of the fragments. CMC-q is an automation software module within Topspin for data acquisition, processing, analysis and quantification of small molecules by NMR spectroscopy. CMC-a is a software tool for interactive, assisted data analysis. It processes all 1D and 2D NMR datasets, performs automated analyses on the different types of NMR experiments and conducts consistency checks. CMC-q uses a ^1^H-NMR spectrum and the corresponding structure file of the fragment for structure verification. Further, using the advanced options, the user can customize and define signals of solvent or known impurities that should not be considered within the structure verification process. In order to confirm the integrity of the fragments, ^1^H-NMR spectrum of the individual fragments with a final approximate concentration of 1 mM in *d*_*6*_-DMSO were acquired. Further, within the drug discovery process, it is also important to have a good idea about the concentration of the ligand in the sample. Several quantitative NMR methods have been described within the literature (Holzgrabe 2010). In general, by NMR the concentration of the substance is determined relative to the known concentration of a standard. We used 1 mM of 1,4-dioxane as an external standard and defined the integral of the signal as *Eretic Reference* (Hoult 2000; Hoult and Richards 2011; Wider and Dreier 2006) in CMC-q. After acquisition of each spectrum, the analysis, consistency check with the structure and concentration determination is performed “on-the-fly” at the spectrometer.

Solubility and retaining integrity of the fragments in the aqueous buffer is an important requirement for performing ligand-detected NMR screening experiments. Especially, hydrolysis-induced degradation in an aqueous buffer may be overlooked if the QC was solely limited to DMSO solutions. For the solubility analysis our typical NMR samples contained ~ 1 mM compound in 50 mM Sodium phosphate buffer at pH 7.4, 150 mM Sodium chloride, 90% H_2_O/10% D_2_O and 1 mM of 3-(trimethylsilyl)propionic-2,2,3,3-d_4_ acid sodium salt (TMSP-Na) added as an internal chemical shift reference and quantification standard.

**Fig. 2:**
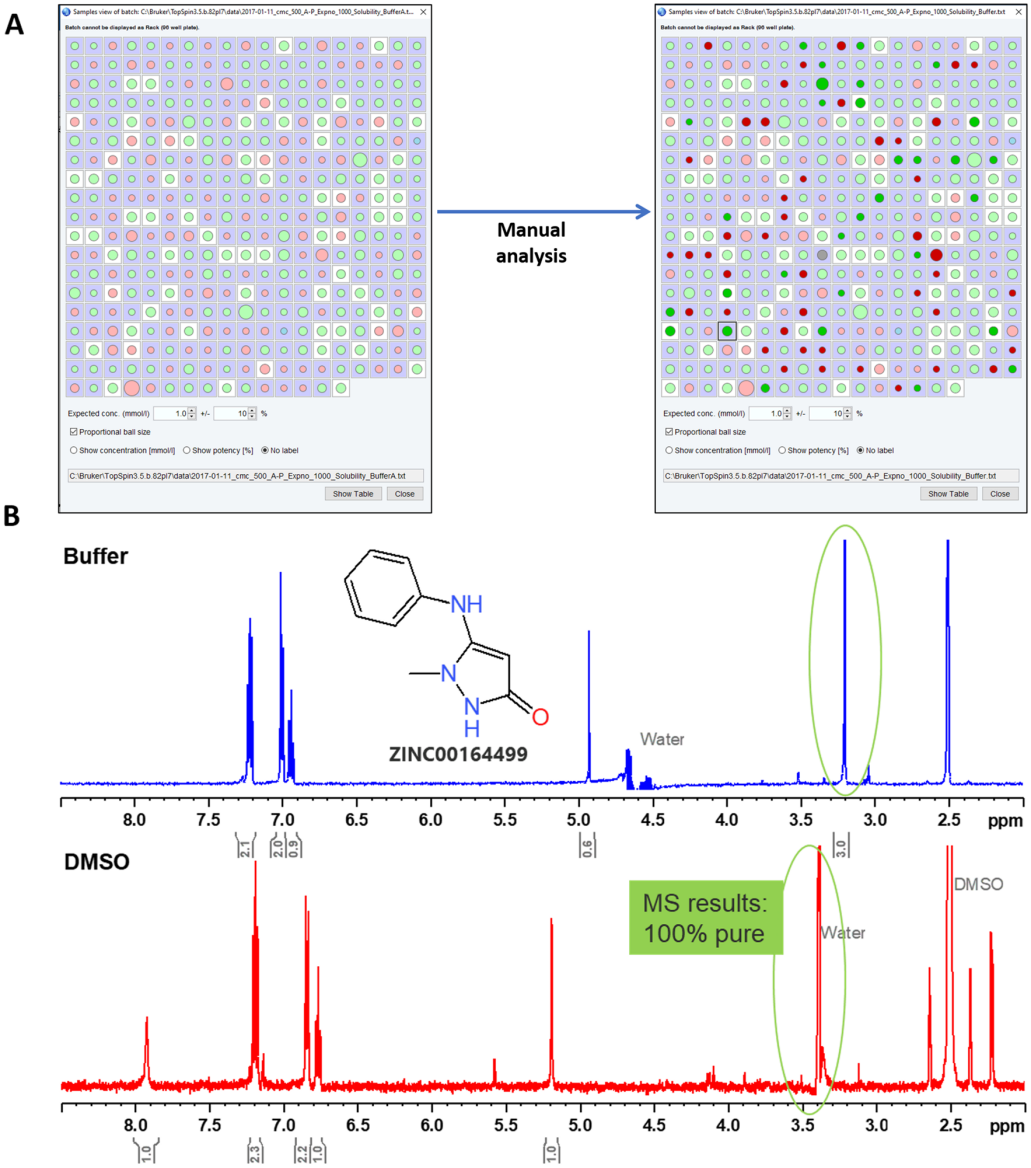
Quality control of the fragments. **a** Screenshot of the graphical display representing the CMC-a based analysis of 358 fragments in a compact form. This window displays the determined concentration and structural consistency (green means consistent; red means inconsistent, blue indicates technical complications, light colors-results from automation, intense colors-results from manual analysis). If the additional option to show the concentration is checked, then the sizes of the displayed circles are proportional to the value. Samples within the range of the expected concentration have a white background. **b**^1^H-NMR spectrum of a fragment acquired in buffer (blue, top) and in *d*_*6*_-DMSO (red, bottom). The proton signal overlapped by the water signal in the red spectrum gets resolved in the blue spectrum

Automated analysis by CMC-a yields a graphical display representing the analysis results of the whole fragment collection (Fig. 2a). This compact representation of the result displays the consistency of the spectra with the structure and also the concentration. For a given fragment a green colored circle indicates “consistent”, a red for “inconsistent” and the size of the circles indicate the concentration. An automated analysis resulted in approximately 65% of the fragments as consistent and 35% as inconsistent both in DMSO and buffer. Further, approximately 60% of the fragments displayed exactly overlapping (consistent/inconsistent) results between the DMSO and buffer measurements. In an effort of identifying false negatives, we performed a manual analysis over a subset of the “inconsistent” fragments and found that approximately 30% additionally turn consistent. Most often either compound signal overlap with the solvent, missed peak picking or incorrect integrals were the reasons for the failure of automated analysis. For example, the methyl group signal of a compound in *d*_*6*_-DMSO appears at 3.4 ppm, however, is not resolved due to a overlap with the water signal (Fig. 2b, bottom). Moreover, this signal (3.2 ppm) is resolved when measured in buffer (Fig. 2b, top). Further, LC–MS data for the compound also revealed that the fragment stock is 100% pure and has the expected molecular mass. However, in an effort to identify the false positives, < 1% of the fragments turned into inconsistent suggesting that the automated analysis performed by CMC-a is robust.

## Integration of methods to eliminate the inconsistent fragments from the library

**Fig. 3:**
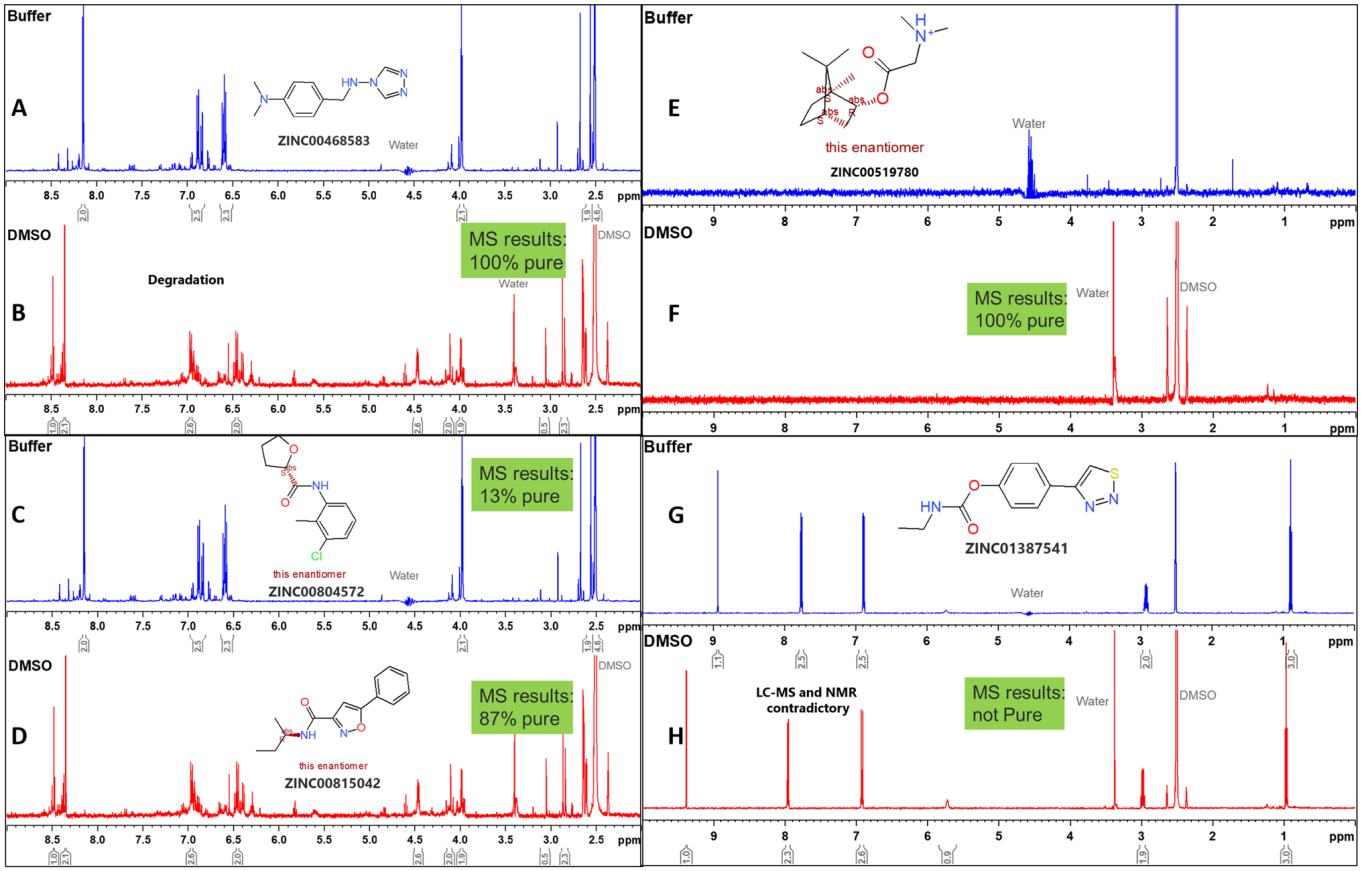
Example spectra of fragments. **a** Degradation of fragment, **b** degradation of fragment as seen in ^1^H-NMR and contradiction between NMR and LC–MS results, (**c**, **d**) impurities, (**e**, **f**) too little compound, (**g**, **h**) passing NMR, but fails in LC–MS

**Fig. 4:**
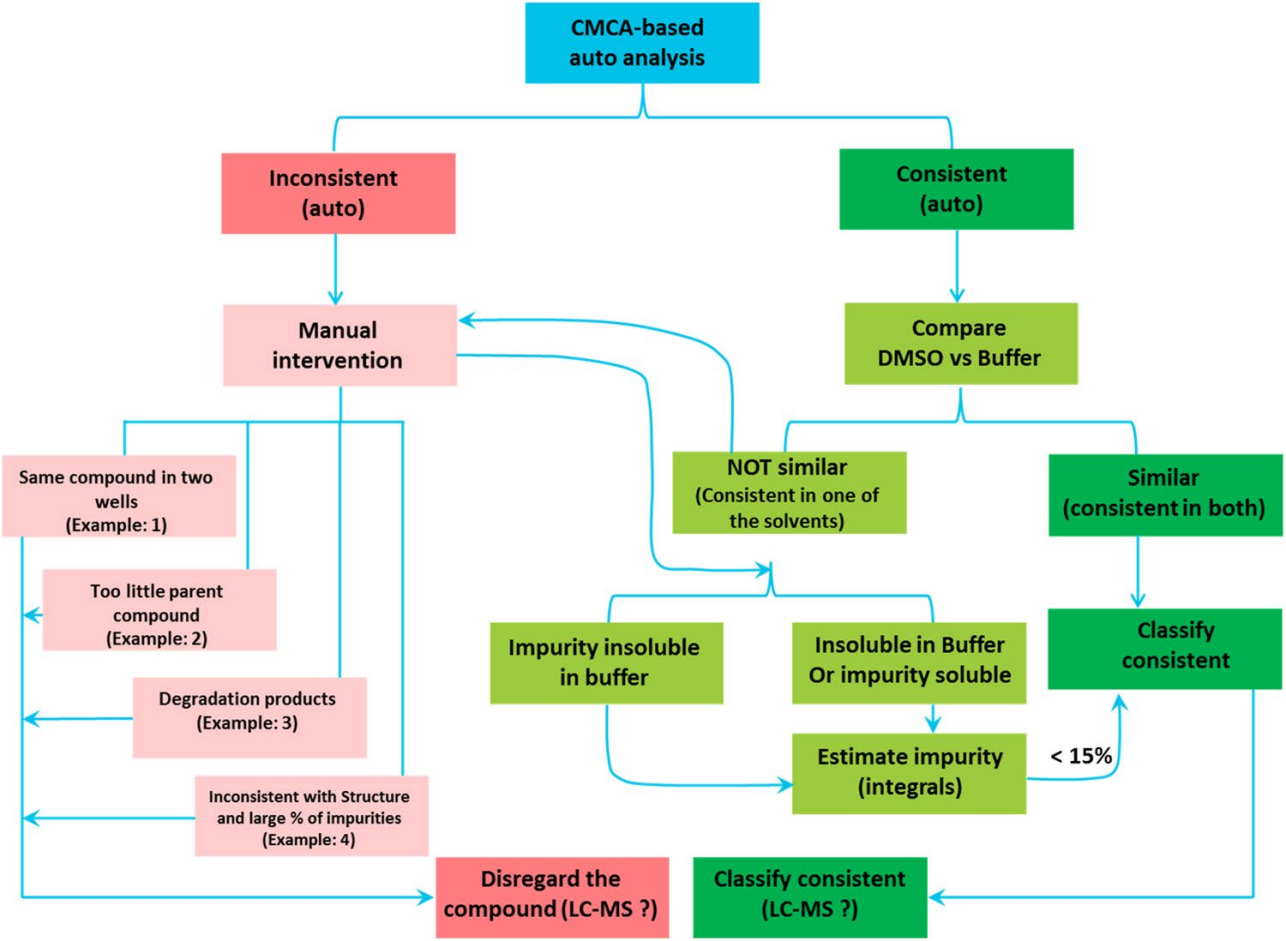
Schematic representation of the workflow during the stringent quality control of the iNEXT fragment library

In general, it is quite common to observe that a significant proportion (between 15 and 40%) of the fragments fail in the QC process (Keseru et al. 2016; Lau et al. 2011). A critical analysis of the QC data obtained from NMR-based automated analysis and LC–MS provides insights into some of the potential causes. We found that most often the reason for QC failure was degradation (Fig. 3a, b), compromised purity, inconsistency with the structure and insolubility or no compound. In couple of instances we observed mixtures of compounds (Fig. 3c, d), though they were located two wells apart in the same plate. This probably would have occurred during the manual assembly of the library. Another challenge we faced was the inconsistency of the results between NMR and LC–MS based QC. LC–MS of the fragment classifies it as not pure (Fig. 3b, g, h), however, the NMR spectra of the same, both in DMSO and also in buffer shows that it is consistent with the structure. Another frequent reason to fail the QC is insolubility of the fragments or no compound or very little compound in the stock (Fig. 3e, f). In general, we adopted an optimized workflow protocol scheme (Fig. 4) in order to streamline the elimination of inconsistent fragments from the library. Initially, an automated analysis is performed by CMC-a, which results in two classifications (consistent-auto; inconsistent-auto). In order, to have a second layer of quality check for those consistent fragments, we then perform a manual assessment of peak patterns between the DMSO and buffer spectra (compare DMSO vs buffer). If they are similar, then they enter into the green zone of the workflow and if not, then will enter into the manual intervention workflow. Approximately, 30% of the fragments were discarded.

## Speed and periodic evaluation of the fragment library

In general, long term stability of the fragment library is an important requirement for performing several screening campaigns. Therefore, periodic evaluation of the fragment library in terms of its quality is indispensable. In order to economically meet these objectives, we took the advantage of the latest state-of-the-art advanced hardware viz., the Bruker robot system *SamplePro Tube™*, with which the liquid sample collection can be filled into the 3 mm NMR-tubes in an automated manner. ^1^H NMR spectra are then collected at 298 K using a 600 MHz Bruker Avance III HD NMR spectrometer equipped with triple resonance 5 mm TCI Prodigy cryogenic probe and a sample changer *SampleJet™*, which can handle more than 500 samples in a go. This together with software tools of Bruker, like CMC-q and CMC-a speeds up the data acquisition and analysis. Typically, for a fragment library comprising of around 782 fragments complete QC could be completed within a span of 3 to 4 days. Maintenance of the library also implies replacing fragments that turn out to be unstable. Storage of the fragment solutions in matrix tubes instead of deep-well plates is therefore preferred.

## Conclusions

The progression of drug discovery within academia has shown significant maturity and has imbibed FBDD as a more commonly utilized approach. Challenges faced in academic FBDD have been significantly overcome with more consortium-based organizations, such as the iNEXT. Within this framework, assembly of a robust fragment library, performing periodic QC and allowing the library to evolve can be a demanding task. We through a set of examples and advanced methods have demonstrated the ease with which one can perform the QC in an academic setting. In general, an integrated choice of methods, viz., NMR, LC–MS together with software assisted validation of a fragment library ensures a relatively high quality of fragments assessed for its integrity, solubility and also stability to endure several screening campaigns.

## Methods

### Sample preparation

The fragments were stored as 50 mM stock solutions in a mixture of 90% *d*_*6*_-DMSO and 10% D_2_O. ^1^H-NMR spectrum of the individual fragments with a final approximate concentration of 1 mM in *d*_*6*_-DMSO /Phosphate buffer pH 7.4 were acquired. The final sample volume was 170 μL with 5% D_2_O as locking solvent in a 3 mm NMR tube.

### NMR spectroscopy

Spectra acquisition was carried out on a Bruker AVIIIHD-500/600 NMR spectrometer. The fully automated acquisition of the data was performed by using Bruker CMC-q software interface within Topspin. The default parameter sets provided within the software were used for acquisition of the data at 298 K. All analysis were performed using Topspin 4.0 with CMC-a addon.

### LC–MS

#### HRMS-instrument

Agilent Technologies 6230 Accurate Mass TOF LC/MS connected to Agilent Technologies HPLC 1260 Series; Column: Thermo Accucore aQ; particle size: 2.6 µM Dimension: 100 × 2.1 mm; Eluent A: H_2_O with 0.1% formic acid Eluent B: MeCN with 0.1% formic acid; conditions: 0.00 min 95% A, 0.2 min 95% A, 2.1 min to 1% A as gradient, 4 min as Stoptime, 1.5 min Posttime for reconstitution. Flow rate: 0.4 ml/min; UV-detection: 220 nm, 254 nm, 450 nm. Injection volume: 1 µl.

For MS analysis compounds were dissolved in 20 mM DMSO and plated on a 384 well plate, 0.5 µl aliquot was taken, diluted with acetonitril/water (1:1, 80 µl) to a concentration of 125 μM and filtered with a Whatman® 384 well plate (0.45 μm hydrophilic PVDF) before measurement. The UV purity was determined based on the absorption at 254 nm.

### Fragment library and the NMR software

The iNEXT fragment library (DSiP-library) can now be purchased from Enamine (https://enamine.net/fragments/plated-libraries/dsi-poised-library). All Bruker software including CMC-a can be downloaded from the Bruker web page.

## Electronic supplementary material

Below is the link to the electronic supplementary material.

Electronic supplementary material 1 (XLSX 285 kb)

Electronic supplementary material 2 (KNWF 21 kb)

## References

[CR1] Baker M (2013). Fragment-based lead discovery grows up. Nat Rev Drug Discov.

[CR2] Bentley M, Doak BC, Mohanty B, Scanlon MJ, Webb GA (2018). Applications of NMR spectroscopy in FBDD. Modern magnetic resonance.

[CR3] Bickerton GR, Paolini GV, Besnard J, Muresan S, Hopkins AL (2012). Quantifying the chemical beauty of drugs. Nat Chem.

[CR4] Brough PA, Aherne W, Barril X, Borgognoni J, Boxall K, Cansfield JE, Cheung KM, Collins I, Davies NG, Drysdale MJ (2008). 4,5-diarylisoxazole Hsp90 chaperone inhibitors: potential therapeutic agents for the treatment of cancer. J Med Chem.

[CR5] Bulfer SL, Jean-Francois FL, Arkin MR (2016) Making FBDD work in Academia. In: Erlanson DA, Jahnke W (eds) Methods and principles in medicinal chemistry. 10.1002/9783527683604.ch10

[CR6] Congreve M, Carr R, Murray C, Jhoti H (2003). A 'rule of three' for fragment-based lead discovery?. Drug Discov Today.

[CR7] Cox OB, Krojer T, Collins P, Monteiro O, Talon R, Bradley A, Fedorov O, Amin J, Marsden BD, Spencer J (2016). A poised fragment library enables rapid synthetic expansion yielding the first reported inhibitors of PHIP(2), an atypical bromodomain. Chem Sci.

[CR8] Dalvit C, Caronni D, Mongelli N, Veronesi M, Vulpetti A (2006). NMR-based quality control approach for the identification of false positives and false negatives in high throughput screening. Curr Drug Discov Technol.

[CR9] Erlanson DA, De Esch IJ, Jahnke W, Johnson CN, Mortenson PN (2020). Fragment-to-lead medicinal chemistry publications in 2018. J Med Chem.

[CR10] Gossert AD, Jahnke W (2016). NMR in drug discovery: a practical guide to identification and validation of ligands interacting with biological macromolecules. Prog Nucl Magn Reson Spectrosc.

[CR11] Holzgrabe U (2010). Quantitative NMR spectroscopy in pharmaceutical applications. Prog Nucl Magn Reson Spectrosc.

[CR12] Hoult DI (2000). The principle of reciprocity in signal strength calculations—a mathematical guide. Concept Magn Res.

[CR13] Hoult DI, Richards RE (2011). The signal-to-noise ratio of the nuclear magnetic resonance experiment. J Magn Reson.

[CR14] Howard S, Berdini V, Boulstridge JA, Carr MG, Cross DM, Curry J, Devine LA, Early TR, Fazal L, Gill AL (2009). Fragment-based discovery of the pyrazol-4-yl urea (AT9283), a multitargeted kinase inhibitor with potent aurora kinase activity. J Med Chem.

[CR15] iNext Consortium (2018). iNEXT: a European facility network to stimulate translational structural biology. FEBS Lett.

[CR16] Jhoti H, Williams G, Rees DC, Murray CW (2013). The 'rule of three' for fragment-based drug discovery: where are we now?. Nat Rev Drug Discov.

[CR17] Keseru GM, Erlanson DA, Ferenczy GG, Hann MM, Murray CW, Pickett SD (2016). Design principles for fragment libraries: maximizing the value of learnings from pharma fragment-based drug discovery (FBDD) programs for use in academia. J Med Chem.

[CR18] Lau WF, Withka JM, Hepworth D, Magee TV, Du YJ, Bakken GA, Miller MD, Hendsch ZS, Thanabal V, Kolodziej SA (2011). Design of a multi-purpose fragment screening library using molecular complexity and orthogonal diversity metrics. J Comput Aided Mol Des.

[CR19] Lepre CA (2011). Practical aspects of NMR-based fragment screening. Methods Enzymol.

[CR20] May PC, Dean RA, Lowe SL, Martenyi F, Sheehan SM, Boggs LN, Monk SA, Mathes BM, Mergott DJ, Watson BM (2011). Robust central reduction of amyloid-beta in humans with an orally available, non-peptidic beta-secretase inhibitor. J Neurosci.

[CR21] Murray CW, Rees DC (2009). The rise of fragment-based drug discovery. Nat Chem.

[CR22] Park CM, Bruncko M, Adickes J, Bauch J, Ding H, Kunzer A, Marsh KC, Nimmer P, Shoemaker AR, Song X (2008). Discovery of an orally bioavailable small molecule inhibitor of prosurvival B-cell lymphoma 2 proteins. J Med Chem.

[CR23] Prochazkova E, Jansa P, Brezinova A, Cechova L, Mertlikova-Kaiserova H, Holy A, Dracinsky M (2012). Compound instability in dimethyl sulphoxide, case studies with 5-aminopyrimidines and the implications for compound storage and screening. Bioorg Med Chem Lett.

[CR24] Rogers D, Hahn M (2010). Extended-connectivity fingerprints. J Chem Inf Model.

[CR25] Shuker SB, Hajduk PJ, Meadows RP, Fesik SW (1996). Discovering high-affinity ligands for proteins: SAR by NMR. Science.

[CR26] Taylor A, Doak BC, Scanlon MJ (2018). Design of a fragment-screening library. Methods Enzymol.

[CR27] Tsai J, Lee JT, Wang W, Zhang J, Cho H, Mamo S, Bremer R, Gillette S, Kong J, Haass NK (2008). Discovery of a selective inhibitor of oncogenic B-Raf kinase with potent antimelanoma activity. Proc Natl Acad Sci USA.

[CR28] Wang YS, Strickland C, Voigt JH, Kennedy ME, Beyer BM, Senior MM, Smith EM, Nechuta TL, Madison VS, Czarniecki M (2010). Application of fragment-based NMR screening, X-ray crystallography, structure-based design, and focused chemical library design to identify novel microM leads for the development of nM BACE-1 (beta-site APP cleaving enzyme 1) inhibitors. J Med Chem.

[CR29] Wider G, Dreier L (2006). Measuring protein concentrations by NMR spectroscopy. J Am Chem Soc.

[CR30] Woodhead AJ, Angove H, Carr MG, Chessari G, Congreve M, Coyle JE, Cosme J, Graham B, Day PJ, Downham R (2010). Discovery of (2,4-dihydroxy-5-isopropylphenyl)-[5-(4-methylpiperazin-1-ylmethyl)-1,3-dihydrois oindol-2-yl]methanone (AT13387), a novel inhibitor of the molecular chaperone Hsp90 by fragment based drug design. J Med Chem.

[CR31] Wyatt PG, Woodhead AJ, Berdini V, Boulstridge JA, Carr MG, Cross DM, Davis DJ, Devine LA, Early TR, Feltell RE (2008). Identification of N-(4-piperidinyl)-4-(2,6-dichlorobenzoylamino)-1H-pyrazole-3-carboxamide (AT7519), a novel cyclin dependent kinase inhibitor using fragment-based X-ray crystallography and structure based drug design. J Med Chem.

[CR32] Zhu Z, Sun ZY, Ye Y, Voigt J, Strickland C, Smith EM, Cumming J, Wang L, Wong J, Wang YS (2010). Discovery of cyclic acylguanidines as highly potent and selective beta-site amyloid cleaving enzyme (BACE) inhibitors: Part I–inhibitor design and validation. J Med Chem.

